# Genetic polymorphisms in *DPF3 *associated with risk of breast cancer and lymph node metastases

**DOI:** 10.1186/1477-3163-4-13

**Published:** 2005-08-19

**Authors:** Carolyn R Hoyal, Stefan Kammerer, Richard B Roth, Richard Reneland, George Marnellos, Marion Kiechle, Ulrike Schwarz-Boeger, Lyn R Griffiths, Florian Ebner, Joachim Rehbock, Matthew R Nelson, Andreas Braun

**Affiliations:** 1 Sequenom, Inc., San Diego, California, USA; 2 Department of Obstetrics & Gynecology, Technical University of Munich, Germany; 3 Genomics Research Centre, School of Health Science, Griffith University Gold Coast, Australia; 4 I. Frauenklinik, Klinikum Innenstadt, University of Munich, Germany

## Abstract

**Background:**

Several studies have identified rare genetic variations responsible for many cases of familial breast cancer but their contribution to total breast cancer incidence is relatively small. More common genetic variations with low penetrance have been postulated to account for a higher proportion of the population risk of breast cancer.

**Methods and Results:**

In an effort to identify genes that influence non-familial breast cancer risk, we tested over 25,000 single nucleotide polymorphisms (SNPs) located within approximately 14,000 genes in a large-scale case-control study in 254 German women with breast cancer and 268 age-matched women without malignant disease. We identified a marker on chromosome 14q24.3-q31.1 that was marginally associated with breast cancer status (OR = 1.5, P = 0.07). Genotypes for this SNP were also significantly associated with indicators of breast cancer severity, including presence of lymph node metastases (*P *= 0.006) and earlier age of onset (*P *= 0.01). The association with breast cancer status was replicated in two independent samples (OR = 1.35, *P *= 0.05). High-density association fine mapping showed that the association spanned about 80 kb of the zinc-finger gene *DPF3 *(also known as *CERD4*). One SNP in intron 1 was found to be more strongly associated with breast cancer status in all three sample collections (OR = 1.6, *P *= 0.003) as well as with increased lymph node metastases (*P *= 0.01) and tumor size (*P *= 0.01).

**Conclusion:**

Polymorphisms in the 5' region of *DPF3 *were associated with increased risk of breast cancer development, lymph node metastases, age of onset, and tumor size in women of European ancestry. This large-scale association study suggests that genetic variation in *DPF3 *contributes to breast cancer susceptibility and severity.

## Background

Breast cancer etiology is a complex process, involving genes in the multiple stages of carcinogenesis, from initial cell cycle dysregulation to metastatic potential [1,2]. Approximately ten percent of breast cancer cases occur within families in which the disease segregates in a Mendelian fashion. *BRCA1 *and *BRCA2 *have been identified to be responsible for a substantial proportion of familial breast cancer [3,4]. Other genes involved in the same DNA double-strand break repair pathway, *TP53 *[5], *ATM *[6], and *PTEN *[7], are also known to contribute to familial cases, but are more rare. Such high penetrance germ line mutations are responsible for less than 10% of all breast cancer cases. However, genetic variation is estimated to contribute approximately 25% to the population risk of breast cancer, likely accounted for by a large number of yet undiscovered common, low penetrance alleles [8,9]. It is possible that these low penetrance markers may be useful in the development of practical prognostic and diagnostic indicators with greater utility in the general population.

Many candidate gene studies have been performed to identify the genes that contribute to risk for sporadic breast cancer [10]. Unfortunately, these efforts have been largely unsuccessful. Some of the more consistently reported candidates include variations in metabolizing enzymes, such as the cytochrome P-450 family [11], N-acetyltransferases [12], and glutathion-S-transferases [13]. The candidate susceptibility allele CHEK2*1110delC was shown to confer an increased breast cancer risk [14,15], which was more recently supported by results obtained in a large case-control study [16]. In an effort to identify novel genes involved in breast cancer susceptibility, we have conducted a large-scale, case-control study using more than 25,000 SNPs located within approximately 14,000 genes. We previously reported the findings on two breast cancer candidates identified in this study [17,18]. Herein, we describe variations in intron 1 of *DPF3 *on chromosome 14q24.3-q31.1 that are associated with increased risk of breast cancer, lymph node metastases, earlier age of diagnosis, and tumor size.

## Methods

### Subjects and Study Design

The participants in the large-scale association study (referred to as the discovery sample) were recruited among patients attending the Frauenklinik Innenstadt, University of Munich, Germany, and comprised 254 breast cancer cases. At the time of assessment, 94 cases (37%) displayed positive lymph node status, and 18 cases (7%) had known distant metastases. Twenty-seven cases (11%) reported having at least one first- or second-degree relative with breast cancer. The median age of diagnosis was 56 yr (range = 23–87 yr). During the same period, 268 controls with a median age of 57 yr (range = 17–88 yr) were recruited from patients with benign disease being seen at the clinic. Controls with a reported family history of breast or ovarian cancer were excluded from the current study. Both parents of each study participant were reported to be of German descent.

The participants in the German replication sample were recruited from the Department of Obstetrics and Gynecology, Technical University of Munich, and consisted of 188 cases and 150 controls. Most breast cancer cases were recruited at pre-operative visits, and the female controls were recruited from healthy individuals or patients with non-malignant diagnoses. Median age of diagnosis for cases was 59 yr (range = 22–87 yr) and median age of controls was 50 yr (range = 19–91 yr). Two participants reported one parent of non-German, Eastern European origin; otherwise both parents were of German descent.

The participants in the Australian replication sample were recruited from the Pathology Department of Gold Coast Hospital or by the Genomics Research Centre, Southport. The collection included 180 breast cancer cases with a median age of diagnosis of 50 yr (range = 24–74 yr). Controls consisted of 180 healthy volunteers without family history of cancer recruited through the Genomics Research Centre. Controls were individually age-matched to cases (± 5 yr) with the median age for controls at 60 yr (range = 28–94 yr).

All subjects involved in our studies signed a written informed consent and the institutional ethics committees of participating institutions approved the experimental protocols.

### SNP Markers, Genotyping, and Resequencing

A set of 25,494 SNPs covering the human genome was selected from a larger collection of 125,799 experimentally validated polymorphisms [19]. This set includes SNPs that are located in gene coding regions (within 10 kb of 13,735 genes annotated in Entrez Gene), have a minor allele frequencies greater than 0.02 (95% have frequencies greater than 0.1), and a median inter-marker spacing of 40 kb. SNP annotation was based upon the NCBI dbSNP database, refSNP build 118 [20]. Genomic annotation was based on NCBI Genome Build 34. Gene annotation was based upon Entrez Gene genes for which NCBI was providing positions on the Mapview FTP site [21].

DNA pools were formed by combining equimolar amounts of each sample as described elsewhere [22,23]. For SNP assays carried out on pooled DNA, 25 ng of DNA was used. All PCR and MassEXTEND™ reactions were conducted using standard conditions [23]. Relative allele frequency estimates were derived from calculations based on the area under the peak of mass spectrometry measurements from four analyte aliquots as described elsewhere [23]. The same procedure was used for individual genotyping except 2.5 ng DNA was used and only one mass spectrometry measurement was taken. Primers used for genotyping are presented in Table 1. Sequencing was performed under standard conditions for MassCLEAVE™ [24] using 5 ng of DNA. For Exon 1, the amplification primers used were 5'-AACGGCAGAGCACATGTAGTAA-3' and 5'-ATATTGAAACCACGCGGAATA-3'. For Exon 2, the amplification primers used were 5'-CTGGGTGTGTTTCAGTCTTCC-3' and 5'-CTGGTTTCCCAGACAAGCTG-3'.

**Table 1:** Primer sequences used in genotyping.

rs8010957	Forward primer	5'-TGC TGG GAT TAT GAG CCA CT-3'
	Reverse primer	5'-GTG TGT CTC CAG TAA AGG GC-3'
	Extend primer	5'-AAA ACT CTG CTA CTG GC-3'
rs4307892	Forward primer	5'-GCA AAA TGC TAG TAA ATG GTG-3'
	Reverse primer	5'-GAA AAA TGG CAA GCC TTC TG-3'
	Extend primer	5'-AGA GCA ATG AAC ACC AAT ATC C-3'

rs1990440	Forward primer	5'-AAG TCA CTA ACC CCA CAC AC-3'
	Reverse primer	5'-CCA GGG TGT GTT CTA ATA CG-3'
	Extend primer	5'-CGT CAG CAA ATG TGT ACC GA-3'

rs4899445	Forward primer	5'-AGG AGA GTC TGC CCA TTT GA-3'
	Reverse primer	5'-AGA AAA CTC ACC TCC CTG AC-3'
	Extend primer	5'-AGC CCT CTC CAG GGC CAT GC-3'

rs4378563	Forward primer	5'-GCC GTG TGC ATA TCC TGA TC-3'
	Reverse primer	5'-TTA TGG CTT CCT CTC CCT AC-3'
	Extend primer	5'-CCT CCA TGC CCT GCT TA-3'

rs12232220	Forward primer	5'-TTA AAA ATA CAA TGA TGG CC-3'
	Reverse primer	5'-TCC CGA CCT CAG GTG ATG TG-3'
	Extend primer	5'-GAT TAC AGG TAT GAG CCA C-3'

### Statistical Methods

Tests of association between disease status and each SNP using pooled DNA were carried out in a similar fashion as explained elsewhere [25]. Sources of measurement variation included pool formation, PCR/mass extension, and chip measurement. When three or more replicate measurements of an allele frequency were available within a model level, the corresponding variance component was estimated from the data. Otherwise, the following historical laboratory averages were used: pool formation = 5.0 × 10^-5^, PCR/mass extension = 1.7 × 10^-4^, and chip measurement = 1.0 × 10^-4^. Tests of association using individual genotypes were carried out using a chi-square test of heterogeneity based on allele and genotype frequencies. Selected tests of association involving contingency tables with rare or missing cells were carried out using Fisher's exact test. The DerSimonian-Laird random effects meta-analysis method [26] was used for the analysis of replication samples to test for the consistency of association while permitting allele frequencies to differ among collections. All tests of allele frequencies involving only replication samples are one-sided, confirming the effect observed in the discovery sample. *P*-values were derived using the log odds of each contrast and their standard errors. Multiple approaches were explored in an effort to identify haplotypes demonstrating a stronger association with disease status than single sites. These included analyses of six SNP haplotypes and subsets thereof using the coalescent theory-based PHASE v2.0 [27] and the score method that relies on the EM algorithm [28]. No attempt was made to correct *P*-values for multiple testing. Rather, *P*-values are provided to compare the relative strength of association from multiple dependent (e.g. SNPs within samples) and independent (e.g. SNPs between samples) sources of information. *P*-values less than 0.05 are referred to as statistically significant.

## Results

**Figure 1:**
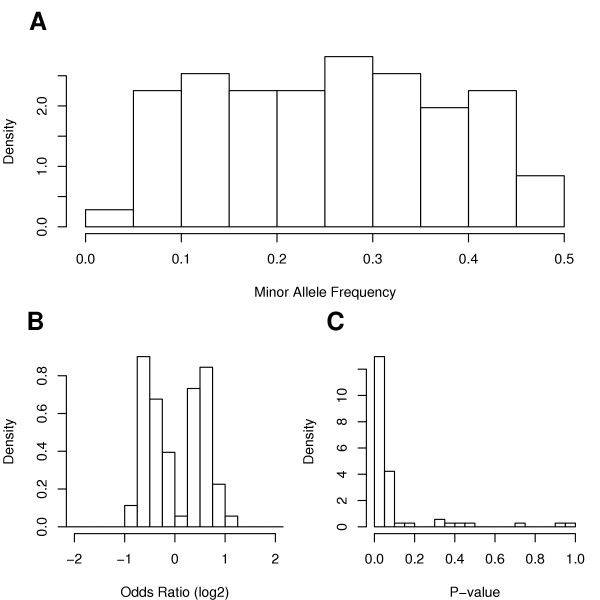
Univariate distributions of summary statistics of the 74 genotyped SNP assays. Empirical densities (histograms) are provided for A) the minor allele frequency in the controls, B) the odds ratios (ORs) on a log2 scale presenting the fold-change comparing allele frequencies between cases and controls, and C) the *P*-values from the tests of association.

SNP markers associated with breast cancer status were identified using a three-phased approach. In the first phase, pools of case and control samples were subjected to a single PCR reaction and primer extension for each of the 25,494 SNP assays. Four aliquots of the extension products were measured. The relative allele frequencies were compared, and 1,619 SNPs (~5%) with the most statistically significant associations were selected to be tested in the second phase. In this phase, allele frequencies were measured in three separate PCR and primer extension reactions using case and control pools, and compared as in the first phase. The 74 most significant SNPs (~5%) from the second phase were selected for individual genotyping in the samples that comprised the case and control pools (Figure 1).

Case-control studies employing tens of thousands of SNPs in a genome-wide approach using liberal selection criteria are expected to yield a high proportion of false positive associations. To determine if the observed association was a true genetic effect, the 74 SNPs were subsequently genotyped in two additional breast cancer case-control collections. After reviewing the results of all three samples, one significant result was observed for a C-to-G SNP, rs1990440, in intron 1 of the DPF3 gene on chromosome 14q24.3-q31.1. The frequency of the G allele in discovery control subjects was 0.08, similar to the NCBI reported average allele frequency [29]. The frequency was increased by 4% in the cases. Table 2 shows the association of rs1990440 with breast cancer in the discovery and two replication collections. Even though this SNP was only marginally associated in the German discovery sample (OR = 1.49, *P *= 0.069), German replication sample (OR = 1.33, *P *= 0.29), and Australian replication sample (OR = 1.36, *P *= 0.22), the estimated effects were consistent and the analysis of all three samples resulted in a combined significance of *P *= 0.016 (OR = 1.40) and a significance of *P *= 0.054 (OR = 1.35) within the replication samples only.

**Table 2:** Distribution of genotype counts and relative allele frequencies of the *DPF3 *polymorphisms^1 ^in breast cancer and control groups for discovery and replication samples.

Study		N^a^	Genotype count (%)			MAF^b^	OR^c^	*P*-value^d^
**rs8010957 (Intron 1 G>A)**			AA	AG	GG	A		
German^2^	Case	235	0 (0)	8 (3)	227 (97)	2%	0.34	0.006
	Control	256	0 (0)	23 (9)	233 (91)	5%		
German^3^	Case	182	0 (0)	9 (5)	173 (95)	2%	0.94	0.912
	Control	134	0 (0)	7 (5)	127 (95)	3%		
Australian	Case	163	0 (0)	5 (3)	158 (97)	2%	0.62	0.407
	Control	164	0 (0)	8 (5)	156 (95)	2%		
				Replication only			0.79	0.270
				Total (all of centres)			0.55	0.058
								
**rs4307892 (Intron 1 G>A)**			AA	AG	GG	A		
German^2^	Case	238	2 (1)	59 (25)	177 (74)	13%	1.56	0.030
	Control	252	1 (0)	44 (17)	213 (83)	9%		
German^3^	Case	185	1 (1)	40 (22)	144 (78)	11%	1.44	0.177
	Control	141	2 (1)	19 (13)	120 (85)	8%		
Australian	Case	173	0 (0)	33 (19)	140 (81)	10%	1.23	0.445
	Control	171	1 (1)	25 (15)	145 (85)	8%		
				Replication only			1.33	0.068
				Total (all of centres)			1.43	0.010
								
**rs1990440^4 ^(Intron 1 C>G)**			GG	GC	CC	G		
German^2^	Case	206	2 (1)	46 (22)	158 (77)	12%	1.49	0.069
	Control	253	2 (1)	39 (15)	212 (84)	8%		
German^3^	Case	191	1 (1)	39 (20)	151 (79)	11%	1.33	0.286
	Control	145	1 (1)	22 (15)	122 (84)	8%		
Australian	Case	179	1 (2)	34 (19)	144 (80)	11%	1.36	0.224
	Control	171	1 (1)	25 (15)	145 (85)	8%		
				Replication only			1.35	0.054
				Total (all of centres)			1.40	0.016
								
**rs4899445 (Intron 1 A>G)**			GG	GA	AA	G		
German^2^	Case	240	2 (1)	48 (20)	190 (79)	11%	1.56	0.045
	Control	257	1 (0)	35 (14)	221 (86)	7%		
German^3^	Case	185	2 (1)	28 (15)	155 (84)	9%	1.72	0.094
	Control	143	0 (0)	15 (10)	128 (90)	5%		
Australian	Case	176	1 (1)	33 (19)	142 (81)	10%	1.47	0.160
	Control	172	1 (1)	22 (13)	149 (87)	7%		
				Replication only			1.56	0.016
				Total (all of centres)			1.56	0.003
								
**rs4378563 (Intron 1 C>T)**			TT	CT	CC	T		
German^2^	Case	235	2 (1)	58 (25)	175 (74)	13%	1.86	0.004
	Control	252	2 (1)	34 (13)	216 (86)	8%		
German^3^	Case	175	1 (1)	30 (17)	144 (82)	9%	1.15	0.624
	Control	131	1 (1)	19 (15)	111 (85)	8%		
Australian	Case	162	1 (1)	30 (19)	131 (81)	10%	1.20	0.506
	Control	167	1 (1)	26 (16)	140 (84)	8%		
				Replication only			1.18	0.210
				Total (all of centres)			1.44	0.027
								
**rs12232220 (Intron 2 G>A)**			AA	AG	GG	A		
German^2^	Case	237	0 (0)	16 (7)	221 (93)	3%	0.55	0.059
	Control	253	1 (0)	28 (11)	224 (89)	6%		
German^3^	Case	186	0 (0)	25 (13)	161 (87)	7%	2.23	0.038
	Control	144	0 (0)	9 (6)	135 (94)	3%		
Australian	Case	166	0 (0)	16 (10)	150 (90)	5%	1.27	0.536
	Control	169	0 (0)	13 (8)	156 (92)	4%		
				Replication only			1.66	0.960
				Total (all of centres)			1.13	0.770

^1^Presented in order respective to position in *DPF3*; ^2^German discovery sample; ^3^German replication sample; ^4^Marker SNP identified in large-scale association study.

^a^Number of subjects with genotypes; ^b^Minor relative allele frequency; ^c^Odds ratio for test comparing allele frequencies between cases and controls; ^d^*P*-value for test comparing allele frequencies between cases and controls.

**Figure 2:**
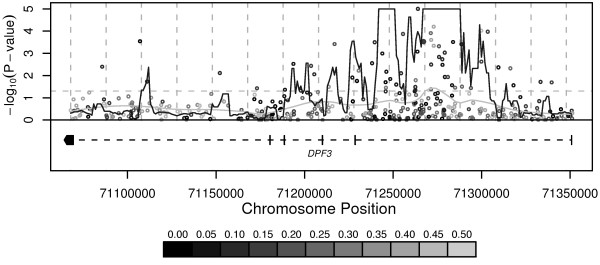
Fine mapping of breast cancer susceptibility on chromosome 14q24.3-q31.1. 395 SNPs in a 250-kb window were compared between pooled cases and controls. The *x*-axis corresponds to the chromosomal position and the *y*-axis to the test *P*-value (shown on the -log_10 _scale). The continuous dark line represents a goodness – of – fit test for excess of significance (compared to the 0.05 proportion expected by chance alone) in a 10-kb sliding window assessed at 1-kb increments. The continuous light grey line is the result of a non-linear smoothing function showing a weighted average of the *P*-values across the region. The shade of each point corresponds to the minor allele frequency of the corresponding SNP in the control sample (see legend below graph). The Entrez Gene annotation for NCBI genome build 34 is included.

To fine map the region of association, we tested an additional 394 SNPs located within the *DPF3 *gene using the discovery case and control pools (Figure 2). We observed that the contiguous region of highest significance extended approximately 65 kb, spanning the 3' region of intron 1 with additional evidence for a 15 kb region that includes exon 2 and a part of intron 2. Using a cleavage assay and mass spectrometry [24], we re-sequenced exons 1 and 2 with their flanking intron sequences in six breast cancer cases and five controls to determine if any additional SNPs with stronger disease association or apparent functional relevance could be discovered. We identified only one SNP in intron 1 that was not publicly annotated (data not shown) and found to have an average allele frequency that did not significantly differ between the case and control pools. No previously described SNPs reside in exon 1 or 2, and no novel SNPs were discovered by our efforts. We selected five SNPs with allele frequencies that differed significantly between case and control pools, roughly distanced 20 kb apart, for genotyping in the discovery and replication samples and for further analysis (Table 1, 2). The SNPs most strongly associated in the discovery and replication samples, rs4307892, rs4899445 and rs4378563, were flanking the original marker SNP and were in strong linkage disequilibrium (all |D'| > 0.9, *r*^2 ^> 0.7). Of the additional SNPs genotyped, rs4899445 demonstrated the most consistent differences between cases and controls, with a slightly larger effect in the discovery sample (OR = 1.56, *P *= 0.045) and a substantially more consistent effect in the German (OR = 1.72, *P *= 0.094) and Australian (OR = 1.47, *P *= 0.16) replication samples (Table 2). The effect of the combined replication sample was significant at the 0.05 level (OR = 1.56, *P *= 0.016) and equal to the estimate from all three samples (OR = 1.56, *P *= 0.003). Analyses of haplotypes consisting of subsets of the six genotyped SNPs did not reveal any haplotype with stronger association than individual SNPs (data not shown).

The data collected on the patients in the German discovery collection included information on family history of breast cancer, age of onset, and disease severity. Further analysis revealed associations between the initial marker SNP, rs1990440, and multiple traits indicative of cancer aggressiveness (Table 3), including lower mean age of diagnosis of breast cancer (*P *= 0.01) and lymph node metastases (*P *= 0.006). Associations with organ metastases (*P *= 0.35) and tumor size (*P *= 0.17) were not statistically significant. The SNP most strongly associated with breast cancer risk across all three samples, rs4899445, was also found to be significantly associated with lymph node metastases (*P *= 0.008), and increased tumor size (*P *= 0.007). Though not statistically significant, the risk allele carriers tended to be younger at age of diagnosis (*P *= 0.35) and to have a higher proportion of breast cancer family history (*P *= 0.13).

**Table 3:** Association of the *DPF3 *polymorphisms with traits of interest in discovery breast cancer cases.

Trait		Genotype Estimates			*P*-value
		AA	AG	GG	
**rs4307892 G>A**	N	N = 2	N = 59	N = 177	
Age of diagnosis (Years)	235	_49.6_54.5 _59.4_	_45.1 _54.6 _60.3_	_51.2 _56.9 _63.5_	0.098^1^
Years since diagnosis	233	_4.6 _8.3 _12.1_	_0.6 _2.8 _5.7_	_0.6 _2.2 _5.9_	0.793^1^
Familial breast cancer history	254	1 (50)	6 (10)	17 (10)	0.255^2^
Tumor size^3^	236	0 (0)	22 (37)	47 (28)	0.318^2^
Lymph node metastases (Yes)	254	2 (100)	23 (39)	46 (26)	0.014^2^
Organ metastases (Yes)	254	0 (0)	6 (10)	10 (6)	0.339^2^
		GG	GC	CC	
**rs1990440 C>G**	N	N = 2	N = 46	N = 158	
Age of diagnosis (Years)	235	_49.6_54.5 _59.4_	_45.0 _51.7 _60.1_	_51.9 _57.4 _65.0_	0.012^1^
Years since diagnosis	233	_4.6 _8.3 _12.1_	_0.4 _3.0 _6.8_	_0.6 _2.2 _5.4_	0.762^1^
Familial breast cancer history	254	1 (50)	6 (13)	15 (9)	0.165^2^
Tumor size^3^	236	0 (0)	20 (43)	44 (30)	0.169^2^
Lymph node metastases (Yes)	254	2 (100)	19 (41)	39 (25)	0.006^2^
Organ metastases (Yes)	254	0 (0)	6 (13)	11 (7)	0.347^2^
		GG	GA	AA	
**rs4899445 A>G**	N	N = 2	N = 48	N = 190	
Age of diagnosis (Years)	235	_49.6_54.5 _59.4_	_46.4 _53.8 _60.6_	_50.6 _56.5 _63.3_	0.350^1^
Years since diagnosis	233	_4.6 _8.3 _12.1_	_0.7 _2.8 _5.7_	_0.6 _2.3 _5.9_	0.743^1^
Familial breast cancer history	254	1 (50)	6 (12)	17 (9)	0.132^2^
Tumor size^3^	236	0 (0)	23 (48)	47 (26)	0.007^2^
Lymph node metastases (Yes)	254	2 (100)	20 (42)	50 (26)	0.008^2^
Organ metastases (Yes)	254	0 (0)	5 (10)	11 (6)	0.415^2^
		TT	TC	CC	
**rs4378563 C>T**	N	N = 2	N = 58	N = 175	
Age of diagnosis (Years)	235	_49.6_54.5 _59.4_	_46.0 _53.8 _60.6_	_51.0 _56.9 _63.4_	0.212^1^
Years since diagnosis	233	_4.6 _8.3 _12.1_	_0.4 _2.5 _6.0_	_0.7 _2.3 _6.0_	0.723^1^
Familial breast cancer history	254	1 (50)	5 (9)	16 (9)	0.274^2^
Tumor size^3^	236	0 (0)	26 (46)	43 (26)	0.011^2^
Lymph node metastases (Yes)	254	2 (100)	23 (40)	46 (26)	0.013^2^
Organ metastases (Yes)	254	0 (0)	6 (10)	10 (6)	0.338^2^

_*a *__*a*_*b *_*c*_represent the lower quartile *a*, the median *b*, and the upper quartile *c *for continuous variables. ^1^Kruskal-Wallis test; ^2^Fisher's exact test; ^3^T ≥ 2 cm.

## Discussion

Here, we report variants in *DPF3*, identified through a large-scale, genome-wide association study, that are associated with increased breast cancer risk, lymph node metastases, decreased age of onset, and increased tumor size. Our study suggests that individuals that carry one or more G alleles of the C-to-G variant rs1990440 have a nominally significant increase in breast cancer risk in comparison with the CC homozygotes. This association was substantiated in two independent collections from Germany and Australia. Fine mapping narrowed down the region of association to approximately 80 kb, spanning the majority of intron 1, exon 2 and a portion of intron 2. Subsequent genotyping of additional SNPs identified an intron 1 SNP, rs4899445, that was more consistently associated with breast cancer status across the three samples (OR = 1.56, *P *= 0.003).

The initial marker in *DPF3 *was one of 74 SNPs identified from a large-scale association study. The estimated effect of this marker was relatively small and would have been discounted had similar effects not been observed in the two replication samples. Even so, the statistical significance observed in the replication samples alone for the marker SNP (rs1990440; *P *= 0.054) or the more significant SNP identified nearby (rs4899445; *P *= 0.016) would not hold up to an experiment-wide type I error rate of 0.05 after correcting for multiple testing. Given that that 74 regions followed up in replication were largely independent on a population level, a conservative Bonferroni correction would require *P*-values to be less than 0.0006 to achieve the stated experiment-wide false positive rate. Indeed, validation of these results will require a larger sample collection. If we were to assume that the true effect is as estimated by the replication samples (OR = 1.5) with a population allele frequency of 6%, aggregate sample sizes on the order of 1,000 cases and controls will be necessary to have adequate power to substantiate the effect of this region on breast cancer risk.

*DPF3 *encodes a zinc finger protein on chromosome 14q24.3-q31.1. Zinc-fingers consist of clusters of cysteines or cysteines and histidines that coordinately bind zinc ions. *DPF3 *is a highly conserved gene homologous to members of the d4 family of zinc-fingers. Previous characterization of the *DPF3 *gene demonstrated that while it is similar to *DPF1 *(*neuro-d4*) and *DPF2 *(*ubi-d4/REQ*), the introns of *DPF3 *are much larger [30]. This fact, in conjunction with the close similarity in the amino acid sequences of the encoded proteins, has led to the suggestion that the increase in intron size of *DPF3 *may result in changes in the regulation of *DPF3 *gene expression. *DPF3 *has a C2H2 domain and a PHD domain, suggesting a role in the direct binding of DNA and in the assembly of large protein complexes. Functional studies of the d4 gene family have suggested that its members participate in regulation of myeloid programmed cell death through the induction of apoptosis [31]. More recently, *DPF3 *has been identified by microarray analysis as a transcription factor that may play a role in the pathogenesis of incipient Alzheimer's disease [32]. Publicly available information on *DPF3 *gene expression is limited; however it has been shown among others to be expressed in both normal and cancerous breast tissue and cell lines [33].

While the current study suggests that variants in *DPF3 *intron 1 are associated with increased breast cancer risk, the possible mechanism by which these variants predispose to breast cancer are purely speculative. The susceptibility allele might be associated with decreased DPF3 activity through the down-regulation of transcription levels or by negatively impacting RNA splicing. This, in turn, may result in a reduction in the ability of DPF3 to induce apoptosis at the cellular level. Apoptosis is a physiological mechanism of cell death that plays an important role in many disease states, including cancer [34]. Imbalance of pro-apoptotic and anti-apoptotic proteins resulting in altered apoptosis may result in tumor development or poor response to adjuvant therapy. Apoptosis requires de-novo synthesis of mRNA and protein, and alterations of DPF3 may lead to reduced response to apoptotic signaling. Additional experimental studies will be required to precisely elucidate the role of DPF3 in breast cancer etiology and progression.

## Conclusion

Our study in women of European ancestry identified significant associations between polymorphisms in *DPF3 *and breast cancer susceptibility, lymph node metastases, earlier age of onset, and tumor size. While three independent samples from the current study support the observed associations, additional studies are needed to verify the results and to further characterize the gene in order to fully understand the role of *DPF3 *in the etiology and progression of breast cancer. These and similar still undiscovered variations of small effect may be useful in the assessment of individual breast cancer risks and in the decisions surrounding patient care.

## Authors' contributions

CRH drafted the manuscript, participated in the development of the SNP marker set and sequencing, as well as in the study design and data analysis. SK participated in the development of the SNP marker set, supervised the operational aspects of the study, and helped to draft the manuscript. RBR was study project leader and participated in data analysis. RR participated in the study design. GM participated in the development of the SNP marker set. MK and USB designed and collected the German replication sample. LRG designed and collected the Australian replication sample. FE and JR designed and collected the German discovery sample. MRN participated in study design and performed the statistical analyses. AB participated in the study design and had the overall scientific responsibility for the study. All authors read and approved the final manuscript.
